# Spin-Dependent Electronic Properties of Bilayer α‑Graphyne Zigzag Nanoribbons: A Density Functional Theory Study

**DOI:** 10.1021/acsomega.6c02506

**Published:** 2026-07-01

**Authors:** Maycon Ericles Macedo Barros, Eduardo Costa Girão, Vincent Meunier, Paloma Vieira Silva

**Affiliations:** † Programa de Pós-graduação em Física, Universidade Federal do Piauí, Teresina, Piauí 64049-550, Brazil; ‡ Departamento de Física, Universidade Federal do Piauí, Teresina, Piauí CEP 64049-550, Brazil; § Department of Engineering Science and Mechanics, Pennsylvania State University, University Park, Pennsylvania 16802-6812, United States

## Abstract

Graphyne-based nanostructures have attracted growing interest due to their unique electronic and mechanical properties arising from a combination of sp and sp^2^ hybridized carbon atoms. Recent advances have enabled the synthesis of various graphyne members, including few-layer films, quantum dots, and nanoribbons, opening new possibilities for nanoscale applications. In this work, we present a first-principles investigation of the spin-dependent structural and electronic properties of bilayer zigzag nanoribbons derived from the α-graphyne lattice. Four stacking arrangements (AA, Ab, AB-α, and AB-β) were considered, all of which result in nonplanar geometries. We demonstrate that bilayer α-graphyne zigzag nanoribbons exhibit a distinct spin-dependent behavior not observed in graphene-based counterparts, where nonplanar geometries preserve and even enhance magnetic ordering. Our results reveal that the electronic behavior of the ribbons is highly sensitive to both the stacking configuration and ribbon width, exhibiting metallic, semiconducting, and half-metallic characteristics. We find that multiple spin-polarized states emerge in the AB-α and AB-β stackings, where different interlayer and intralayer magnetic alignments lead to distinct electronic behaviors. In particular, we identify stacking-dependent half-metallic states, highlighting a mechanism for intrinsic spin filtering in these systems. Additionally, we show that an external electric field can effectively modulate the band structure of the ribbons, inducing a semiconductor-to-metal or half-metallic transition, depending on the field direction and intensity. These findings provide key insights into the tunability of spin-dependent electronic properties in α-graphyne bilayer systems, with potential implications for spintronic and nanoelectronic device applications.

## Graphical abstract

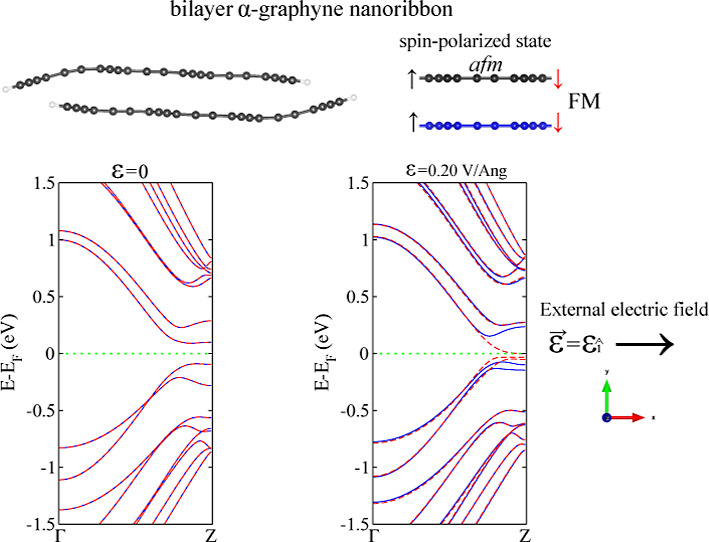

## Introduction

Carbon’s ability to form a wide range of allotropes with diverse physical and chemical properties is largely attributed to its capacity to adopt sp, sp^2^, and sp^3^ hybridizations. For example, the natural 3D allotropes, graphite and diamond, arise from sp^2^ and sp^3^ hybridizations, respectively, resulting in markedly different electronic behaviors: semimetallic in graphite and insulating in diamond. In today’s science, graphene is the most prominent member of the nanocarbon family.^1^ This honeycomb lattice is composed entirely of sp^2^-hybridized carbon atoms, and it is known for its exceptional electrical and thermal conductivity, which are relevant to various practical applications, including electronics, sensors, and optoelectronics.^2,3^ Beyond graphene, there are also 2D carbon allotropes with a combination of carbon atoms with distinct hybridizations, such as graphyne (GY) sheets. Proposed by Baughman et al. in 1984,^4^ GYs can be conceptualized as graphene derivatives in which acetylenic linkages (containing triple carbon–carbon bonds) are inserted into the hexagonal lattice, resulting in structures composed of both sp and sp^2^ carbon atoms. There are various GY lattices, such as α-, β-, and γ-GY (which are popular examples in the literature), characterized by different ratios and arrangements of acetylenic linkages.^5,6^ Notably, the coexistence of sp and sp^2^ hybridized carbon atoms gives rise to diverse electronic and structural properties, making GYs highly attractive for applications in nanoelectronics, catalysis, and energy storage.^7,8^

The GY family gained prominence after the first experimental observation of graphdiyne (GDY),^9^ a structure similar to γ-GY that features two-unit-long acetylenic chains. Since then, several new GY structures have been proposed, and various members have been successfully synthesized.^10–14^ Similar to graphene-based systems, several studies have demonstrated that the properties of 2D GY layers can be tuned through quantum confinement and boundary effects when they adopt nanoribbon-type geometries. For example, α- and β-GY nanoribbons with armchair edges are nonmagnetic and exhibit semiconducting behavior, whereas those with zigzag edges display spin-polarized (SP) states and can be either metallic or semiconducting depending on their edge chirality and their width.^15–17^ In contrast, nanoribbons based on the γ-GY lattice are all semiconducting, regardless of their edge chirality or width.^17,18^ In addition, the electronic properties of nanoribbons can be modulated by applying an external electric field. Previous studies have shown that the band gap decreases as the strength of an externally applied electric field increases.^17^ In spin-polarized ribbons, a semiconductor-to-half-metallic transition has been reported when the applied electric field exceeds a critical threshold.^15,17,19^

Another strategy to tune the electronic structure of GY monolayers is to stack them in bilayer configurations. Inspired by the rich chemical and physical properties of layered graphene, several studies have investigated how the stacking order influences the properties of 2D bilayer GY systems, revealing novel features beyond those of their monolayer counterparts.^20–23^ In this context, exploring interlayer interactions in bilayer GY nanoribbon (BGyNR) systems is particularly attractive, as quantum confinement and edge effects can strongly influence the resulting properties. Similar investigations have already been conducted for graphene-based nanoribbons.^24–29^ For instance, zigzag-edged graphene nanoribbons (ZGNRs) can adopt either AA or Bernal AB stacking, with the latter allowing for two distinct arrangements. The spin-dependent electronic properties of these systems are highly sensitive to the stacking configuration, leading to a variety of electronic behaviors.^24–29^ To the best of our knowledge, only one previous study has addressed bilayer GY nanoribbons, specifically based on the α-GY structure.^30^ While the authors reported structural details for nanoribbons with different stacking configurations, they did not consider possible spin-polarized states. Their analysis focused primarily on the thermoelectric properties of nanoribbons with a specific stacking arrangement, motivated by their band gap characteristics.^30^ This gap in the literature highlights an open and promising research direction, particularly in the field of spintronics, given the potential spin-dependent properties of such systems.

In this work, we present a first-principles investigation of the geometric and electronic properties of bilayer zigzag nanoribbons based on the α-GY lattice (α-BZGyNRs). Using density functional theory (DFT), we examine the influence of different stacking arrangements and spin configurations on the behavior of these nanoribbons. All stacking modes are found to adopt nonplanar geometries, with the AB-α configuration exhibiting pronounced curvature near the edges. Notably, the nonplanarity does not suppress magnetic coupling, which gives rise to distinct spin-polarized states in the AB-α and AB-β stacking configurations. Our results show that the electronic behavior of the α-BZGyNRs is highly sensitive to both the stacking configuration and the magnetic alignment, leading to a range of electronic characteristics. Furthermore, we studied the effect of applying an external electric field on the electronic properties of the α-BZGyNRs.

### Methods

Structural optimizations and electronic structure calculations were performed within the framework of density functional theory (DFT),^31,32^ as implemented in the SIESTA code.^33^ Core electrons were described using norm-conserving Troullier–Martins pseudopotentials,^34^ while valence wave functions were expanded in a double-ζ polarized (DZP) basis set. The exchange–correlation functional was treated using the standard generalized gradient approximation (GGA) as parametrized by Perdew, Burke, and Ernzerhof (PBE).^35^ In particular, considering the underestimated electronic band gap values expected from the PBE functional, additional electronic band structure calculations were also performed with the Heyd–Scuseria–Ernzerhof hybrid functional (HSE06)^36,37^ using the HONPAS package.^38,39^ Real-space integrations were carried out with a mesh cutoff of 400 Ry, and the Brillouin zone (BZ) was sampled with 32 Monkhorst–Pack *k*-points for all α-BZGyNRs studied. van der Waals (vdW) interactions were accounted for by using Grimme’s DFT-D2 dispersion correction method.^40^ Structural relaxations were performed until the residual atomic forces and maximum stress were below 0.01 eV/Å and 0.1 GPa, respectively. To prevent interactions between periodic images, a vacuum region of 20 Å was introduced along all nonperiodic directions.

## Results and Discussion

### Studied Structures

As in 2D bilayer α-graphyne (α-BLGY), the bilayer zigzag α-GY nanoribbons (α-BZGyNRs) can exhibit different stacking configurations, which are easily recognized by the relative position of particular sets of atoms on the stacked layers. In the upper-left part of Figure 1a, these atoms are labeled using capital (lower case) letters A and B (a and b) for the atoms with sp^2^ (sp) hybridization, as highlighted by pink-colored letters. To distinguish the various stacking configurations in α-BLGY, we adopt a notation similar to that used for bilayer graphene, since both lattices are bipartite.^20,41^ However, bipartition in α-GY is defined for both types of carbon hybridization, sp and sp^2^, which results in several possible bilayer stacking arrangements. In this work, we considered four stacking orders labeled as AA, Ab, AB-α, and AB-β, as illustrated in Figure 1a, where the two layers are indicated by different colors. In AA stacking, each carbon atom in the top layer is positioned directly above its equivalent atom in the bottom layer. In AB stacking, the A atoms of one layer are aligned over the B atoms of the other layer, while in Ab stacking, the A atoms of one layer lie above the b atoms of the other, following the terminology introduced in ref 20. As we move from α-BLGY to α-BZGyNRs, two variants are considered for the AB stacking: α and β. These variants are defined based on the sublattice type of the edge atoms in one layer relative to the other. The relevant atoms lie along the same line perpendicular to the nanoribbon axis. In the AB-α configuration, the corresponding edge atoms belong to the same sublattice in both layers, whereas they belong to opposite sublattices in AB-β. Examples of these stacking configurations are shown in Figure 1a for the 4-α-BZGyNR case. The width of the α-BZGyNR is denoted by *n*, corresponding to the number of zigzag lines from edge to edge, as illustrated for the 4-α-BZGyNR with AA stacking in Figure 1a.

**Figure 1:**
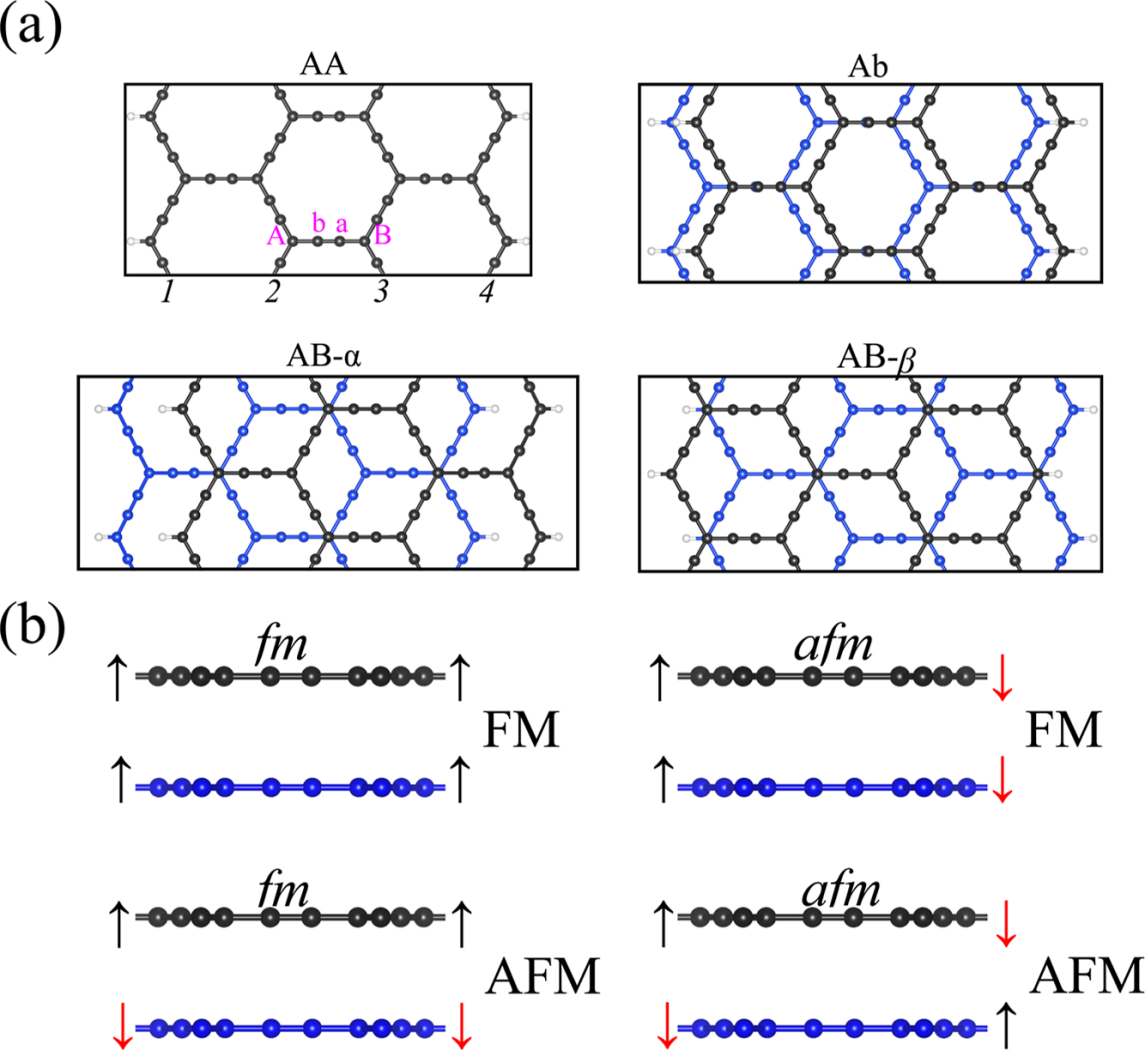
(a) Representation of the stacking configurations of the starting simulation setup for the α-BZGyNR systems: AA, Ab, AB-α, and AB-β. The black layer corresponds to the upper layer, and the blue layer corresponds to the bottom layer. Hydrogen atoms are denoted by white spheres. (b) Initial configurations for the spin-polarized states of the α-BZGyNRs. Interlayer (intralayer) coupling can be either ferromagnetic [FM­(fm)] or antiferromagnetic [AFM­(afm)].

We studied α-BZGyNRs with widths ranging from *n* = 2 to *n* = 8, where the width of the corresponding monolayer α-ZGyNR varies from 10.16 Å (for *n* = 2) to 46.75 Å (for *n* = 8). Here, *n* denotes the number of zigzag lines along the ribbon’s width. Considering the different stacking configurations and ribbon widths, we refer to each system as *n*-α-BZGyNR-*stacking mode*. For example, Figure 1a shows the initial atomic structure for the 4-α-BZGyNR in the AA, Ab, AB-α, and AB-β configurations. We investigated four spin-polarized charge configurations for each stacking mode, as illustrated by the cross-sectional views in Figure 1b. To distinguish between intralayer and interlayer magnetic coupling, we use capital letters to denote interlayer coupling and lowercase letters for intralayer coupling. The FM–fm configuration (upper-left diagram in Figure 1b) corresponds to ferromagnetic coupling both within and between the layers. In the AFM–fm configuration (bottom-left diagram), the layers are ferromagnetically ordered individually but antiferromagnetically coupled to each other. The remaining two configurations, FM–afm and AFM–afm, shown in the upper- and bottom-right diagrams of Figure 1b, feature antiferromagnetic coupling within each layer but with ferromagnetic and antiferromagnetic coupling between the layers, respectively.

For the structural optimizations, the interlayer distance of the α-BZGyNRs was initially set to 3.0 Å, a value chosen to be close to the van der Waals distance (vdW) in carbon-based systems.^10,42^ Since spin polarization in ZGyNRs is known to be localized at the edges, the different spin-polarized states were initialized by assigning specific initial charges to the edge atoms in both layers at the beginning of each self-consistent calculation, allowing the system’s charge to converge properly. For each initial spin configuration, we fully relaxed the atomic structures, allowing the system to evolve toward a stable spin-polarized state. For the AA stacking, with the exception of the 2-α-BZGyNR case, all initial spin-polarized guesses converged to a nonpolarized (NP) electronic distribution. For the 2-α-BZGyNR-AA case, the only initial guess that converged to a nontrivial spin-paired distribution was the AFM–afm configuration. In the Ab stacking, regardless of the ribbon width, all the spin-polarized initial configurations resulted in a nonpolarized state after full relaxation and convergence. For the AB stacking, in both the α and β alignments, the AFM–fm and FM–fm initial guesses did not result in stable solutions for the 2-α-BZGyNR system. However, for wider ribbons (*n* ≥ 3), all the initial configurations resulted in stable spin-polarized states after full relaxation and convergence, except the AFM–fm state for the 3-α-BZGyNR-AB-α ribbon.

### Binding Energy and Structural Details

Before discussing electronic properties in detail, we first examine the stability of α-BZGyNRs by reporting their binding energy, *E*_BE_, calculated as the difference between the total energy of the α-BZGyNR and the energies of the individual layers, which is defined according to the following equation:

1 $$E_{BE}=\left(E_{\alpha -BZGyNR}-\sum_{i}E_{layer-ghost}^{i}\right)/N$$

where *E*_α–BZGyNR_ is the total energy of the α-BZGyNR, *E*_layer‑ghost_^*i*^ is the total energy of the *i*th isolated monolayer ribbon, and *N* is the number of atoms in the unit cell. To avoid basis set superposition errors (BSSE),^43^ the calculation of *E*_layer‑ghost_^*i*^ is performed in the same geometric setup as the α-BZGyNR system, including the basis functions of the other layer (ghost orbitals) but omitting the atomic potentials from those atoms.

Figure 2a shows the *E*_BE_ values as a function of ribbon width *n* for α-BZGyNRs in AA, Ab, AB-α, and AB-β stacking configurations. For the AB-α, AB-β, and AA (only for *n* = 2 ribbon) stackings, we calculated the energy difference between each spin-polarized and the corresponding nonpolarized state. These data are shown in Tables S1, S2, and S3 of the Supporting Information. For the AA case, only the AFM–afm state emerges for the *n* = 2 nanoribbon, which is the lowest energy state, while the ground state is NP for the AA-stacked α-BZGyNRs with *n* > 2. For all α-BZGyNRs with AB-α and AB-β stackings, the FM–afm configuration corresponds to the system’s ground state. Accordingly, the reported *E*_BE_ values in Figure 2a correspond to the spin-polarized ground states of each stacking configuration, namely, the FM–afm state for AB-α and AB-β stackings, the AFM–afm state for the 2-α-BZGyNR-AA system, and NP for the other cases.

**Figure 2:**
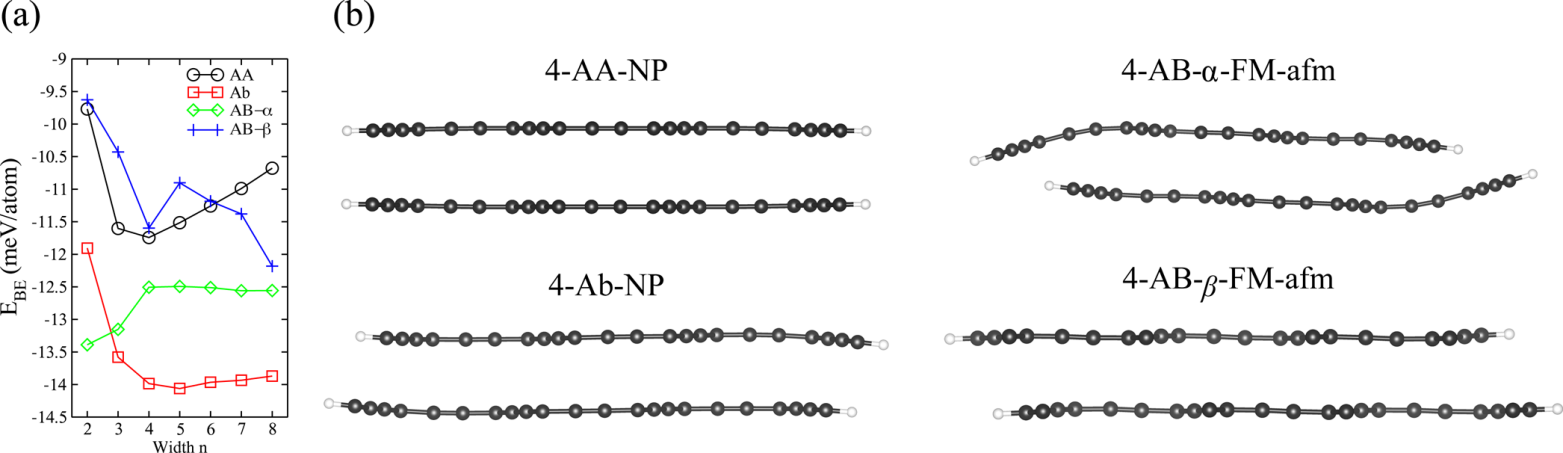
(a) Binding energy (*E*_BE_) of α-BZGyNRs as a function of ribbon width *n*, after full structural relaxation. (b) Optimized atomic structures of the 4-α-BZGyNR system for AA, Ab, AB-α, and AB-β stacking configurations. In the AB-α and AB-β cases, the structures correspond to the FM–afm ground state.

Comparing the different stacking modes, the Ab stacking is generally the most stable for a given ribbon width *n*, with the exception of the 2-α-BZGyNR system, for which the AB-α configuration is the ground state. When comparing the AB-α and AB-β configurations, for a given width *n*, the AB-α mode is more stable, but the energy difference between the two stacking modes decreases as the width increases, except for the 5-α-BZGyNR case. We interpret this last exception as the *n* ≤ 4 systems showing a different trend than the *n* > 4 systems due to their narrower width ranges, so that their edge effects play a more important role than in narrow systems. On the other hand, while having intermediate stability for narrow systems, the AA stacking is revealed to be the least stable configuration for the widest setups, as we gradually recover 2D-like behaviors as we increase the system’s width.

We now turn to the structural properties of the α-BZGyNRs. After full geometry optimization, starting from a planar configuration, we observe that all ribbons become nonplanar, regardless of their width or stacking mode. In Figure 2b, we present the cross-section views for the atomic structures of the 4-α-BZGyNR systems for the states with the smallest total energies in the AA, Ab, AB-α, and AB-β stackings. Therefore, we show the α and β alignments in their FM–afm ground states. Nevertheless, the atomic structures of all AB-stacked systems do not depend significantly on the spin-polarized configuration. As shown in Figure 2b, the ribbons exhibit edge bending caused by the attractive interaction between the edges of the upper and lower layers. The AA case only changes slightly from the perfect planar form, while the AB-α stacking exhibits the strongest curvature effects, followed by the AB-β system. To quantify the out-of-plane distortions, we calculate the interlayer distances at the center (*d*_center_) and at the edges (*d*_edge_) of the ribbons as a function of width. In AA stacking, the *d*_center_ value is ≈3.35 Å for the 2-α-BZGyNR system, while for the *n*-α-BZGyNRs with *n* ≥ 3, *d*_center_ is ≈3.38 Å, which is very close to the corresponding value in the 2D AA bilayer system, which is ≈3.39 Å. At the edge region, we identify two types of atomic distances: (1) between sp^2^–sp^2^ atoms and (2) between sp–sp atoms. For the first distance type, we identify two well-defined values: ≈3.19 Å and ≈3.29 Å. For the second type, the distance is ≈3.34 Å. In the Ab stacking mode, the distances between layers involve (1) sp–sp^2^ and (2) sp–sp atom pairs, both measuring approximately ≈3.14 Å. In AB-α stacking, only the *d*_center_ value is properly defined, due to the strong edge-to-edge interaction between the layers. However, both *d*_edge_ and *d*_center_ values are well-defined for the AB-β stacking configuration. Independent of the alignment type, the distance between the layers is very close to the value in the corresponding 2D AB bilayer configuration, which is 3.03 Å, except for the 2-α-BZGyNR system, for which the values *d*_center_ and *d*_edge_ are 3.18 Å and 3.12 Å, respectively, for AB-α and AB-β stacking modes.

It is relevant to compare the results obtained here with those of bilayer graphene-based nanoribbons (BZGNRs). Bilayer ZGNRs are nonplanar only in the AA stacking and in certain cases of AB-α stacking. We calculated the values of *d*_center_ and *d*_edge_ for *n*-BZGNRs with *n* ranging from 1 to 10, using the same methods employed for the α-BZGyNRs. For BZGNRs with AA stacking, the values of *d*_center_ and *d*_edge_ increase and decrease with increasing width of the BZGNR, respectively. In AB-α stacking, *d*_center_ remains nearly constant, while *d*_edge_ decreases as the width increases. In both stackings, *d*_edge_ is always smaller than *d*_center_ for a given width, which leads to a nonplanar structure. These results are in qualitative agreement with previous studies.^24,26,28,29^ The difference between the results of α-BZGyNRs and BZGNRs is the fact that the *d*_center_ and *d*_edge_ values in α-BZGyNRs do not significantly vary with ribbon width, and the edge curvature is more pronounced in the α-BZGyNRs. These effects can be attributed to two main factors: (1) the presence of acetylenic links, which render the graphyne sheet more flexible than the graphene sheet, and (2) stronger vdW interactions in the α-BZGyNRs.^44^ The first factor can be understood by the presence of large hexagonal pores in the α-graphyne sheet. The second is due to the high electronic charge associated with the insertion of the sp-hybridized atoms.^44^ Previous studies on tubular α-graphyne-based configurations have shown that the combination of lattice flexibility and high electron density can lead to exotic structural deformations.^45,46^ Therefore, the porosity of the graphyne layers reduces the overall steric repulsion between the π-electron clouds compared to pristine graphene, allowing for a closer proximity between layers, as well as the inherent flexibility of graphyne-like structures enabling atomic rearrangements at the edges of each layer of the α-BZGyNR system. This explains why the interlayer distance quickly tends to recover the equilibrium value of the corresponding 2D configuration, resulting in highly curved edge regions in nanoribbons with type α alignment. This unusual interlayer proximity suggests differential electronic coupling, which will be detailed in the Electronic Properties section.

### Electronic Properties

Before moving on to the discussion of the electronic properties, we first examine the nature of the interlayer interactions in the α-BZGyNR-AB systems. We analyzed the spatial distribution of the charge density difference defined as

2 $$\Delta \rho =\rho_{\alpha -BZGyNR}-\rho_{\alpha -ZGyNR}^{upper}-\rho_{\alpha -ZGyNR}^{lower}$$

where ρ_α–BZGyNR_ is the electronic charge density of the α-BZGyNR and ρ_α‑ZGyNR_^upper^ (ρ_α‑ZGyNR_^lower^) is the electronic charge density of the upper monolayer ribbon (lower). The ρ_α‑ZGyNR_^upper^ (ρ_α‑ZGyNR_^lower^) density is calculated using the α-BZGyNR system’s geometry to avoid BSSE. In this setup, ghost orbitals from the lower (upper) layer are included, but the atomic potentials of those atoms are excluded, as in the calculation of binding energies. We also analyzed the spin-resolved projected density of states (PDOS) associated with the p_z_ orbital of each atom in each layer. These results are shown in Figure 3a,b for the 4-AB system in the α and β configurations, respectively. In Δρ plot, the accumulation and depletion of electrons are represented by the yellow and cyan surfaces, respectively.

**Figure 3:**
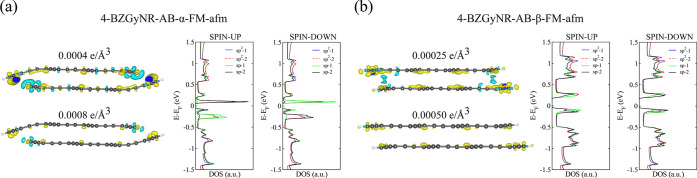
Charge density difference (Δρ) and spin-resolved density of states (PDOS) projected onto the p_z_ orbitals of the sp and sp^2^ atoms of each layer of the 4-α-BZGyNR-AB-α and 4-α-BZGyNR-AB-β systems in the FM–afm state.

In the α alignment, the Δρ plot shows a redistribution of electronic density characterized by charge depletion (holes) and accumulation (electrons) localized over the edge atoms, particularly for the isosurface value of 0.0004 *e*Å^–3^. The holes are distributed over the edges closer to the center of the bilayer, namely, the right-hand (left-hand) side edge for the upper (lower) layer, with minor contributions from the internal atoms from the system. The dominant position of the electrons is on the outermost edges of both layers. However, for the isosurface value of 0.0008 *e*Å^–3^, this charge accumulation and depletion become negligible, especially in the internal part of the system, suggesting that the π interaction between layers is relatively weak and does not exhibit, for example, the pancake-shaped bonding character, characterized by a persistent electronic density “bridge” between layers.^47,48^ As shown in the PDOS plot, the sp^2^ atoms of both layers contribute similarly to the frontier states, since the PDOS is spin-degenerate and layer-degenerate. On the other hand, the sp-hybridized atoms exhibit localized features with a clear interlayer spin correlation. For the energy values at +0.10 eV, −0.09 eV, and −0.25 eV in the PDOS, the spin-up states in one layer coincide with spin-down states in the opposite layer. This indicates an interlayer coupling where π states are correlated across the layers with opposite spin alignment. This behavior suggests that the interlayer interaction is not only governed by π–π orbital overlap but also involves spin-dependent coupling, particularly in regions associated with sp-hybridized carbon atoms.

Figure 3b shows the Δρ and PDOS plot for the 4-AB-β system. For this structure, the charge redistribution is significantly more subtle, requiring lower isosurface values (0.00025 *e*Å^–3^) to be visualized. We observed that there is no strong accumulation of electrons between the layers, while holes are distributed over the edges closer to the center of the bilayer. At higher isosurface values, these features become more negligible. The PDOS reveals a trend similar to the α structure for both sp and sp^2^ atoms, although the spin splitting per layer for sp-hybridized atoms is significantly reduced. Similar behavior to the α and β cases has been observed for the other α-BZGyNRs. These results suggest that the interlayer interaction is primarily driven by dispersion forces, supplemented by π–π coupling that is more localized and spin-dependent at the sp-carbon sites. Consequently, the relatively short interlayer distances (<3.20 Å) arise from the interplay between the inherent flexibility of the α-graphyne lattice, dispersion-driven stacking, and moderate, spatially heterogeneous π–π interactions.

We begin our discussion of electronic properties by revisiting the electronic structure of monolayer ZGyNRs. These nanoribbons are known to exhibit three possible electronic configurations: NP, FM, and AFM. In the upper panel of Figure 4, we present the electronic band structures of these states for a ZGyNR nanostructure with width *n* = 4 (4-ZGyNR), where full blue (dashed red) lines represent spin-up (-down) bands. Similar to zigzag-edged graphene nanoribbons, the NP state of ZGyNRs exhibits nearly flat degenerate bands at the Fermi level (*E*_F_), with corresponding states located at the edges of the structure.^49,50^ This is illustrated in the left-hand side of the lower panel of Figure 4, where the local density of states (LDOS) near *E*_F_ is shown for the NP configuration. These edge states originate from the bipartite nature of the lattice and can be understood as chiral symmetry-protected states, analogous to those found in zigzag graphene nanoribbons.^51,52^ However, once magnetization is included, chiral symmetry is broken, and the edge states are suppressed.^15^

**Figure 4:**
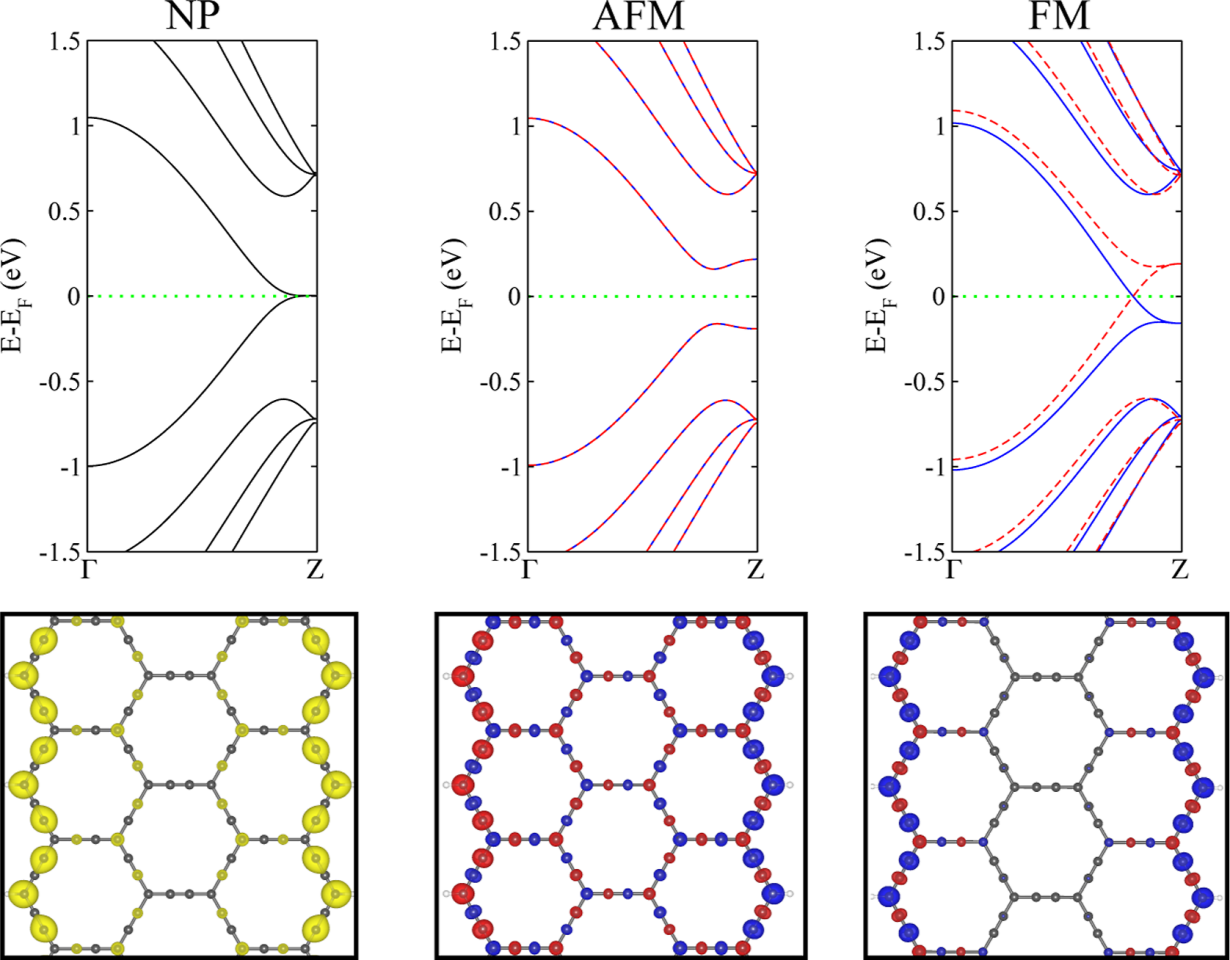
(Upper panel): Electronic band structures of the 4-ZGyNR in nonpolarized (NP), ,antiferromagnetic (AFM), and ferromagnetic (FM) configurations. The spin-up (-down) levels are represented by full-blue (dashed-red) lines. (Lower panel): LDOS plot for states around the Fermi level for NP configuration and spin density plot for the AFM and FM configurations, with excess spin-up (spin-down) charge represented by blue (red) surfaces.

The AFM spin distribution corresponds to the ground state of ZGyNRs and exhibits a semiconducting behavior with spin-degenerate sets of bands, as shown in the top panel of Figure 4. In the lower panel of Figure 4, we further show the corresponding difference between the spin-up and -down components of the electronic density, here called spin density, in the AFM and FM states, with blue (red) clouds representing excess spin-up (-down) charges. For the AFM state, we note that the opposite spin densities can be superimposed on top of each other by a mirror plane operation along the ribbon axis, resulting in a state with a zero total magnetic moment. On the right-hand side of the structure, the hydrogenated sp^2^ atoms and the sp edge atoms closest to the next sp^2^ atoms carry the most intense spin polarization, namely, spin-down as the majority spin. The distribution is inverted (regarding spin-up and -down) on the opposite edge. In contrast, the FM configuration has the same majority spin on both edges, which breaks the symmetry between the spin-up and spin-down electronic charges. Such a symmetry breaking is evidenced by the spin-split (nondegenerate) bands observed in the upper panel of Figure 4. We observed that spin polarization is strongly localized at the edges of the ribbon in the FM state, while internal atoms carry a more significant contribution to spin polarization in the AFM case, although the largest amplitude is at the edges.

Turning to the bilayer structures, to the best of our knowledge, there is only one study on bilayer nanoribbons based on the graphyne structures.^30^ On the other hand, there is extensive literature on bilayer zigzag graphene nanoribbons. Previous theoretical works have shown that the electronic structure of BZGNRs depends strongly on the stacking mode. For instance, the AA and AB-α stackings feature no spin-polarized states, unlike the AB-β stacking.^24,26,28,29^ Most of these studies have focused on the electronic properties of BZGNRs. Only recently, Asano and Nakamura^29^ discussed the origin of magnetism suppression in these bilayer configurations. They observed that in the AA and AB-α stackings, the edge atoms of the bilayer interact strongly, leading to nonplanar structures. In these cases, the edge character of the frontier states is destroyed, and these states shift away from the Fermi level, turning the nonmagnetic configuration into the system’s ground state. Conversely, the AB-β stacking remains planar, preserving the edge character of the frontier states in the bilayer configuration.^29^

We now move to the electronic properties of α-BZGyNRs, initially without considering the spin degree of freedom. In Figure 5 we show the electronic band structures and density of states (DOS) for the 2-α-BZGyNR in AA, Ab, AB-α, and AB-β stacking modes. The AA case has a pair of dispersive bands crossing the *E*_F_; consequently, we observe an energy interval with nonzero constant DOS values around *E*_F_. These frontier states are spread over the entire structure, as shown by the plot of local DOS, in contrast to the behavior observed in the monolayer counterpart, as previously discussed.^15^ In the DOS spectrum, multiple van Hove singularities (vHs) are observed, symmetrically distributed around the Fermi level, but not close to the *E*_F_. Regarding potential device applications, we anticipate that these vHs could play a significant role in charge transport through molecular systems when 2-α-BZGyNR-AA is used as an electrode. This assumption is based on the idea that alignment between molecular levels and vHs peaks can strongly influence the conductance profile.^53,54^ Note that these vHs differ significantly from those of the monolayer counterpart (even disregarding the central one at the *E*_F_).

**Figure 5:**
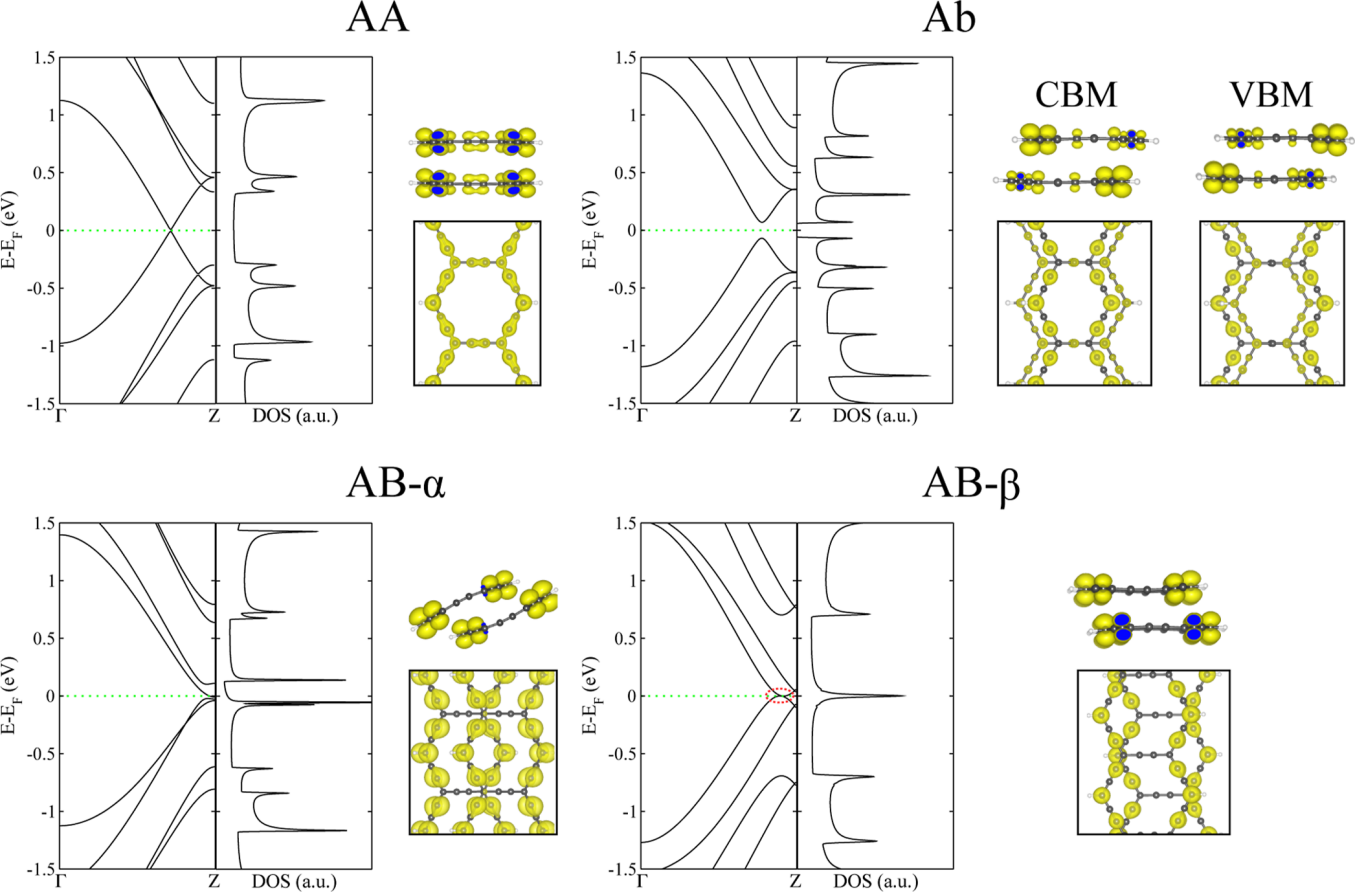
Electronic band structures and total (DOS) and local (LDOS) density of states plots of the 2-α-BZGyNR in AA, Ab, AB-α, and AB-β stacking modes. For 2-α-BZGyNR-AA, -AB-α, and -AB-β systems, the LDOS plot is for states around the Fermi level, while that for 2-α-BZGyNR-Ab is for VBM and CBM states. In the LDOS plot, the yellow regions represent the electron density distribution, while the blue regions appear due to the cross-section of the surface in the visualization procedure and have no physical meaning.

These vHs are also present in the DOS profile of the 2-α-BZGyNR system in Ab stacking. This α-BZGyNR has semiconductor properties, with a band gap of 270 meV at a point intermediate between the Γ and *Z* points of the ribbon’s BZ. We note that the valence band maximum (VBM) and conduction band minimum (CBM) correspond to the first vHs peaks at ± 0.14 eV. Figure 5 displays the LDOS plots for the VBM and CBM of the 2-α-BZGyNR-Ab system, showing that both frontier states are spatially delocalized, but in a complementary fashion. For the CBM state, the LDOS is distributed over the edges closer to the center of the bilayer, namely, the left-hand (right-hand) side edge for the upper (lower) layer. The outermost edges of both layers carry a smaller contribution to the CBM. Conversely, the VBM spreads on the outer edges of the system: the right-hand (left-hand) side edge for the upper (lower) layer, with a smaller contribution from the inner edges. The second pair of vHs exhibits peaks nearly twice as intense as those from the first pair (originating from the VBM/CBM), similar to what we found for the 2-α-BZGyNR-AA system. These more intense peaks are associated with the degeneracy of bands at ±0.45 eV and ±0.32 eV for 2-α-BZGyNR-AA and 2-α-BZGyNR-Ab, respectively, and their spatial distribution is strongly localized at the α-BZGyNRs edges (not shown here). This is similar to the frontier *E* = *E*_F_ states in monolayer α-ZGyNRs, where the frontier bands meet each other. These results indicate that both AA and Ab stacking modes shift the ZGyNR’s edge states away from *E*_F_ in a similar manner while yielding a nearly constant DOS for AA stacking and opening a band gap for the Ab configuration. Such shifts of the bands at *Z* can be viewed as resulting from a hybridization between states originating from the individual layers, with the opening being correlated to the strength of the interlayer orbital hopping. In the Ab case, this can be further interpreted in terms of the breaking of the edge-to-edge symmetry for a given layer, as explicitly seen from charge redistribution effects. For a qualitative analysis, we look at the Mulliken population of the sp^2^ carbons at the edges of the two layers. The charge on the atoms in the innermost bilayer edges decreases by 0.003 *e* relative to those of the isolated monolayer, while the atoms on the outermost edges receive 0.009 *e*. Such a symmetry breaking is analogous to what is observed for zigzag GNRs subjected to a transverse electric field parallel to the molecular plane, which shifts the edge states relative to the *E*_F_.^55^ On the other hand, the gap opening in similar systems with Ab stacking is related to the symmetry breaking of the system’s two sublattices.^46^

The AB-α and AB-β configurations exhibit metallic character but with distinct features. In these stackings, we note the edge states (originating from the *Z* point of the BZ) are not significantly shifted away from *E*_F_. This can be understood from the details of the atomic structures, as only a limited set of atoms is located below sites from the opposite layer, namely, one sp^2^ edge atom per layer per unit cell. In this way, interlayer hybridizations occur to a weaker extent in these configurations. In the AB-α stacking, the frontier bands still show low dispersion near the Fermi level but are nondegenerate (different from the monolayer counterpart). As a result, the DOS plot displays two prominent peaks close to *E*_F_ in negative energy values. This can also be related to the fact that two of the four bilayer edges in the AB-α case are significantly far away from the atoms in the opposite layer, which contributes to preserving their localized character. In contrast, two frontier bands are degenerate at *E*_F_ for the AB-β stacking in a *k*-point before the *Z* edge of the BZ, as highlighted by the dashed red ellipse in Figure 5, leading to an intense peak at *E*_F_ in the DOS. Furthermore, we note that the difference between the frontier states of the AB-α and AB-β in terms of their band branches can be rationalized by comparing them with their 2D counterparts. Bilayer graphene and α-graphyne in the AB stackings show parabolic bands at *E*_F_,^20^ which are somehow similar to those of the AB-β system close to the Fermi level, as the two component ribbons in the AB-β case are almost centered over each other. In contrast, the component ribbons are laterally shifted to each other to a larger extent in AB-α, making the set of interlayer interactions less similar to those of the 2D counterpart so that AB-α′s frontier bands feature less resemblance to the 2D than the AB-β system. To gain more insight into the spatial distribution of these states, in Figure 5, we plot the LDOS for a 0.10 eV energy range around the Fermi level for both stacking modes. These electronic states are highly localized at the edges of the structures, analogous to their monolayer counterparts.^15^ This edge-state localization and the proximity of the peaks to the Fermi level suggest that these stacking modes may potentially support spin-polarized states. Moreover, we note that AB-β stacking has vHs at the ±0.70 eV energies, which are associated with extrema values of some branches of the band structure. Similar to the AA and Ab configurations, the AB-β stacking may play an important role as an electrode in electronic transport setups due to its symmetrical distribution in relation to *E*_F_.^53,54^

The *n*-α-BZGyNRs with *n* > 2 in AA and Ab stacking modes present electronic band structures similar to those of 2-α-BZGyNR. Regarding the band gap values in the Ab configuration, we find a nonmonotonic dependence on the ribbon width. For narrower nanoribbons, the band gap increases, reaching a maximum of 310 meV for the 5-α-BZGyNR-Ab system, and then decreases for increasing width, reaching 110 meV for 8-α-BZGyNR-Ab. A similar trend was reported by Rodrigues et al.,^30^ although the authors identified the 4-α-BZGyNR-Ab as the critical width corresponding to the maximum band gap. The discrepancy between our result and that reported by Rodrigues et al.^30^ might be due to the van der Waals correction adopted by the authors,^56^ which differs from that applied in the present work.^40^ However, the difference in band gap values between the 4-α-BZGyNR-Ab and 5-α-BZGyNR-Ab systems studied here is approximately 40 meV, a value comparable to that observed between the 3-α-BZGyNR-Ab and 4-α-BZGyNR-Ab cases reported by Rodrigues et al.^30^ Such a semiconductor nature of α-BZGyNRs-Ab systems is interesting for thermoelectric applications, for instance, as explored in ref 30, and can be controlled by the stacking type. Moving to AB stacking, we observe little difference in the electronic band structures for different α-BZGyNRs widths. In Figure 6, we show the electronic band structures and DOS for the 4-α-BZGyNR in the AB-α and AB-β cases. In the AB-α configuration, two frontier bands become flat and degenerate near *E*_F_, while the lower conduction band crosses the Fermi level around its minimum value, resulting in a sharp DOS peak at *E*_F_, as shown in Figure 6. In the AB-β case, all frontier bands become degenerate at the *Z* point of the BZ. This behavior of the bands for the AB-α and AB-β configurations is observed for all systems with width *n* > 2 (not shown here).

**Figure 6:**
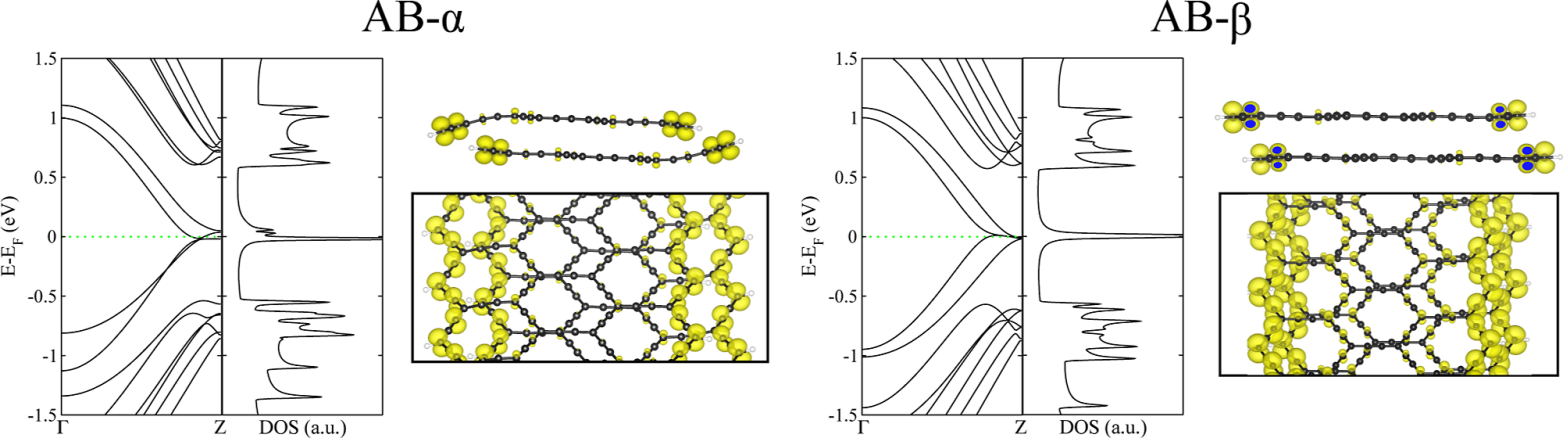
Electronic band structures and total (DOS) and local (LDOS) density of states plots of the 4-α-BZGyNR in AB-α and AB-β stacking modes. The LDOS plot is for states in the close vicinity of the Fermi level. In the LDOS plot, the yellow regions represent the electron density distribution, while the blue regions appear due to the cross-section of the surface in the visualization procedure and have no physical meaning.

### Spin-Polarized States

In Figure 6, we show the LDOS for frontier states of both types of AB stacking of 4-α-BZGyNR. As we can see, these electronic levels are edge states, similar to those of the 2-α-BZGyNR system. Considering that edge states in carbon-based nanoribbons usually give rise to nontrivial spin distributions,^15,16,49,50,57^ we search for spin-polarized configurations in the AB stacking setups of the α and β types, as described in the section “Studied Structures”. Inspired by Santos et al.,^26^ who reported a spin-polarized configuration for the AA stacking of BZGNRs, we also simulate different spin-polarized states for AA stacking. In Figure 7, we present the electronic band structures for the spin-polarized configurations of the 2-α-BZGyNR systems, with spin-up (-down) bands represented by full-blue (dashed-red) lines. As previously discussed, from the four spin-polarization configurations tested for the initial guess of the self-consistency cycle, only AFM–afm emerged as a self-consistent solution for the AA stacking, as well as both AFM–afm and FM–afm configurations for the two AB stacking types.

**Figure 7:**
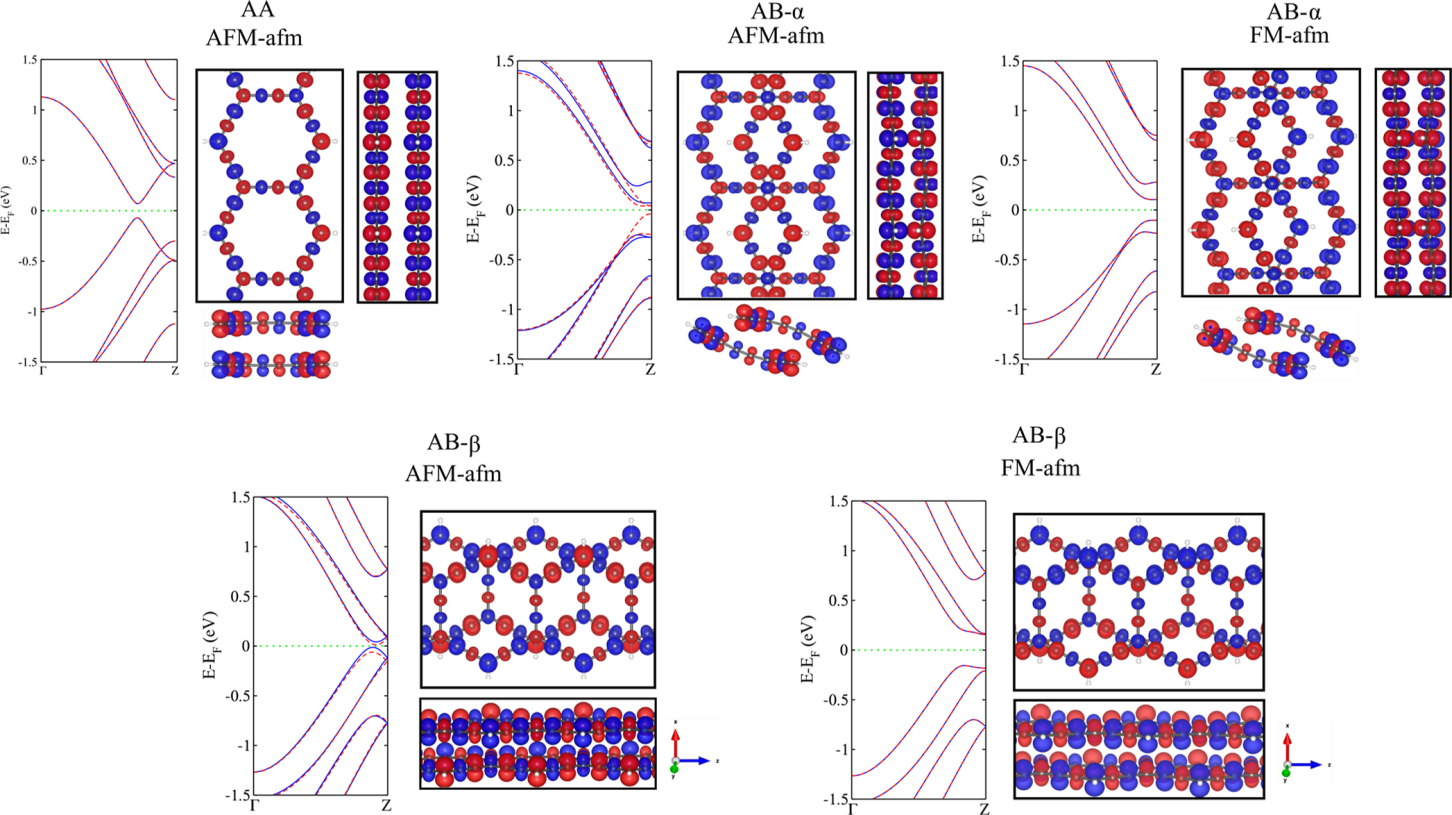
Electronic band structure and spin density plot for 2-α-BZGyNR-AA and -AB systems in their spin-polarized configurations. The spin-up (-down) levels are represented by full-blue (dashed-red) lines, and excess spin-up (spin-down) charge is represented by blue (red) surfaces in the spin density plot.

In the 2-α-BZGyNR-AA case, the spin-up and spin-down bands are degenerate, resulting from its symmetry-related zero total magnetic moment. On the right side of the band structure, the corresponding spin density is shown from different perspectives, where blue (red) clouds represent an excess of spin-up (spin-down) charges. The opposite spin densities exhibit mirror symmetry: they can be superimposed through a mirror-plane operation along the ribbon axis and perpendicular to the bilayer, as in the monolayer system, and also through a mirror-plane operation parallel to the bilayer. The main difference compared to the nonpolarized state is the opening of a 140 meV band gap at the *E*_F_, which can be associated with the breaking of sublattice symmetry (as different sublattices show opposite majority spins), as observed in many other cases.^15,30,46,58^ For *n*-α-BZGyNRs with *n* > 2, no spin-polarized configurations are observed for the AA and Ab stackings.

Regarding the 2-α-BZGyNR-AB structures, both the AFM–afm and FM–afm states exhibit semiconducting behavior for both AB-α and AB-β stacking types, with the FM–afm states displaying wider band gaps and spin-degenerate bands, arising from the symmetry of the spin-resolved charge distribution. For the AFM–afm state, we observe that the bands are significantly spin-split only near the Fermi level, especially for the AB-α. In such a state, the spin distribution over the individual layers is somewhat preserved, being similar to that of an AFM single-layer ribbon (which features spin-degenerated bands). On the other hand, the outer (same for the inner) edges in this AFM–afm distribution have the same spin orientation, as shown by the spin density plot in Figure 7, which breaks the spin-up versus spin-down balance and splits the bands. Hence, since the spin-up/spin-down balance is broken just by the interlayer relative alignment, it only results in a mild splitting of the spin-resolved bands. The band gap of the 2-α-BZGyNR-AB-α system in the AFM–afm state occurs between two spin-down bands, with a value of approximately 79 meV, whereas a much larger (302 meV) band gap is associated with the spin-up bands. For the AFM–afm state of 2-α-BZGyNR-AB-β, the band gap is very narrow, approximately 25 meV, with the VBM and CBM states arising from opposite spin components (up/down for the VBM/CBM). There is another gap from the lower spin-down valence band to the next upper spin-up conduction band of 100 meV.

For *n*-α-BZGyNRs with *n* > 2, all tested spin-polarized configurations give rise to a self-consistent configuration, except for the AFM–fm state for 3-α-BZGyNR-AB-α. This is consistent with previous knowledge on carbon systems such as zigzag graphene and α-graphyne nanoribbons, in which the energy stabilization mechanism due to spin polarization becomes stronger for wider systems.^15,49^ In Figure 8, we show the values of the band gap as a function of width for AFM–afm, AFM–fm, and FM–afm configurations of the α-BZGyNR-AB-α and α-BZGyNR-AB-β systems. In the AFM–afm case of the AB-β system, we note a smaller spin-splitting of the bands compared to that of the AB-α system. This is expected due to the component layers of AB-β being almost laterally aligned to each other, which only slightly breaks the symmetry of the four bilayer edges in two sets of two edges. In the AB-α counterpart, the edge misalignment along the system’s orthogonal direction is larger, so a larger spin splitting is observed for the AB-α case. The FM–afm state of both stackings is semiconducting, with band gaps of 210 and 310 meV for AB-α and AB-β stacking of the 2-α-BZGyNR system, respectively. Independent of the different structural details between the AB-α and AB-β systems, no spin-up/spin-down symmetry is broken in the FM–afm configurations, so they feature spin-degenerated sets of bands.

**Figure 8:**
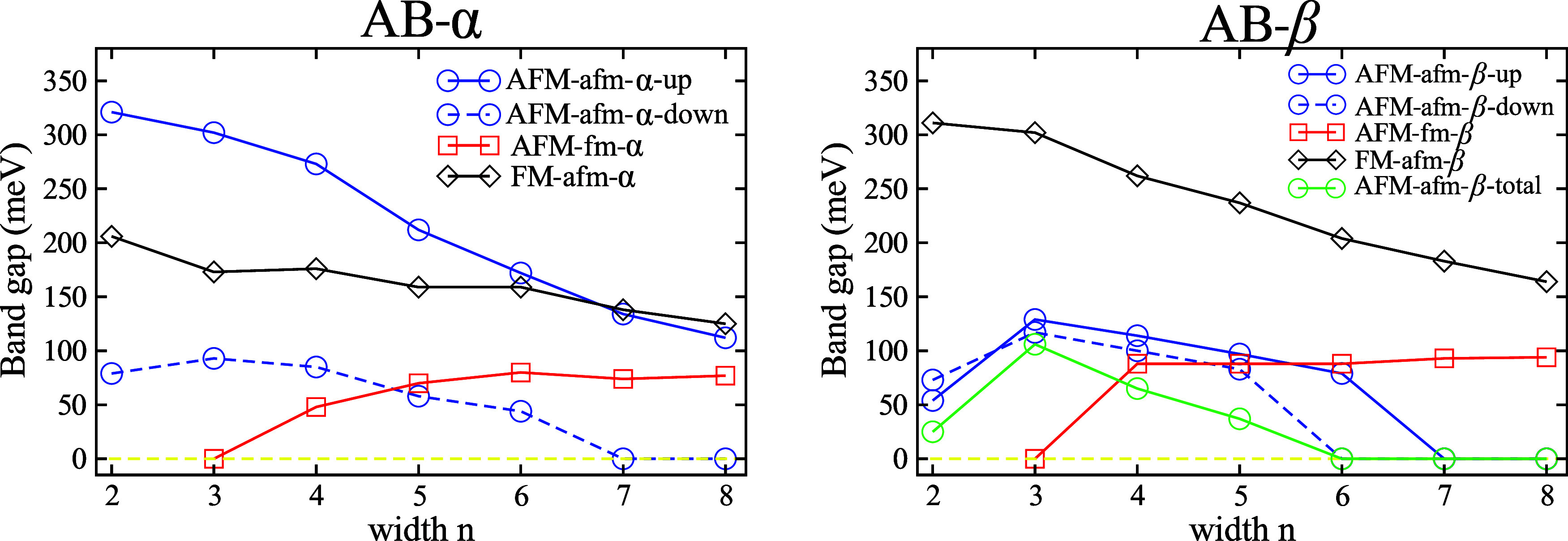
Band gap (in meV) as a function of width *n* for AFM–afm, AFM–fm, and FM–afm configurations of α-BZGyNR-AB-α and -β systems. In each graph, the blue, red, and black color line corresponds to the AFM–afm, AFM–fm, and FM–afm configurations, respectively. In the case of the AFM–afm state, the solid blue line (dashed) represents the band gap for the spin-up (-down) component. The solid green line in the graph for AB-β systems represents the value of the total band gap (between a spin-down and spin-up band). In each graph, the dashed yellow line marks the band gap zero value.

In Figure 9, we show the electronic band structures and spin density plot for all spin-polarized configurations of the AB-α and AB-β stacking modes of the 4-α-BZGyNR-AB system. For the AB-α system, the AFM–afm state exhibits a semiconducting character, with a total band gap of 85 meV arising from the spin-down bands, which are the majority spin at the outer edges. However, a larger band gap of 273 meV is observed for the spin-up bands (majority spin of the inner edges). This electronic behavior is consistent across all *n*-α-BZGyNR-AB-α systems with 2 ≤ *n* ≤ 6, as also summarized in Figure 8. Remarkably, the band gap closes for the spin-down component in the 7- and 8-α-BZGyNR systems, indicating a half-metallic behavior.

**Figure 9:**
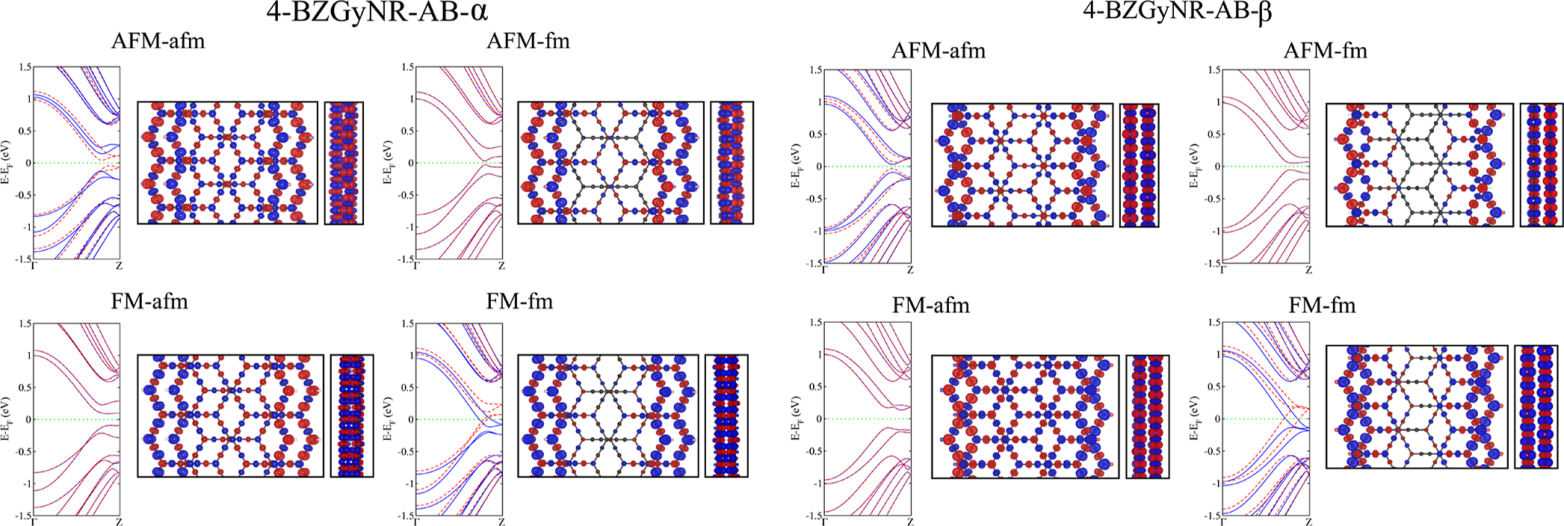
Electronic band structures and spin density plots for AFM–afm, AFM–fm, FM–afm, and FM–fm configurations of the 4-α-BZGyNR-AB-α and -β systems. The spin-up (-down) levels are represented by full-blue (dashed-red) lines, and excess spin-up (spin-down) charge is represented by blue (red) surfaces in the spin density plot.

As in the 2-α-BZGyNR-AB-α ribbon, the FM–afm state of the 4-α-BZGyNR-AB-α system exhibits degenerate spin bands and semiconducting behavior. A similar behavior is observed for the 4-α-BZGyNR-AB-α case in the AFM–fm configuration, but with the FM–afm configuration displaying a larger band gap, as shown in Figure 9. The corresponding band gap values for these spin-polarized configurations are also shown in Figure 8. We observe that the band gap of the FM–afm state decreases with increasing ribbon width, except for the 4-α-BZGyNR system, which is slightly wider than the 3-α-BZGyNR case. In contrast, the band gap of the AFM–fm state increases from *n* = 4 to *n* = 6 and shows very small oscillations for *n* = 7 and *n* = 8, as shown in Figure 8. The FM–fm configuration exhibits metallic behavior, with nondegenerate spin bands of both components crossing the Fermi level. A similar trend is observed for wider α-BZGyNRs (not shown here). Regarding spin densities, we observe that when the intralayer magnetic coupling is of the fm (afm) type, the spin polarization is strongly concentrated at the edges (it has a significant contribution from the internal atoms, although its main contribution comes from the edges), with a profile similar to that of the monolayer (see Figure 4).

A similar behavior is observed for the electronic states of the 4-α-BZGyNR-AB-β ribbon compared to 4-α-BZGyNR-AB-α. For instance, the AFM–fm and FM–afm configurations are spin-degenerate band semiconductors, with a modulation of their band gap values: 88 and 262 meV, respectively. Figure 8 also shows the band gap values of the AFM–afm, AFM–fm, and FM–afm states for all the *n*-α-BZGyNRs-AB-β studied ribbons (*n* from 2 to 8). The AFM–fm configuration corresponds to a stable electronic distribution for the 3-α-BZGyNR ribbon; however, unlike the other ribbons in this family, it exhibits a metallic behavior. As shown in Figure 8, the AFM–fm band gap remains approximately 88 meV for the 4-, 5-, and 6-α-BZGyNR systems and slightly increases to 93 and 94 meV for the 7- and 8-α-BZGyNR ribbons, respectively. On the other hand, the band gap of the FM–afm state decreases with increasing ribbon width, similarly to what is observed in the AB-α family. However, there is a significant gap modulation relative to the other stacking type, as the FM–afm gap of the α-BZGyNR-AB-β systems is consistently wider than those of the α-BZGyNR-AB-α counterparts (see Figure 8). The AFM–afm configuration is semiconducting for ribbons with widths in the 2 ≤ *n* ≤ 5 range, with the VBM and CBM featuring opposite spin contributions. The 6-α-BZGyNR system is half-metallic, with a band gap of 79 meV for the spin-up component, while the spin-down bands cross *E*_F_. Conversely, the 7- and 8-α-BZGyNR systems display metallic behavior. Also, as in the *n* = 2 case, the spin-splitting of the bands is much less marked than in the α counterpart. Finally, the FM–fm spin configuration of 4-α-BZGyNR-AB-β also exhibits metallic character, similar to that of the 4-α-BZGyNR-AB-α ribbon (the same for wider systems).

To address the well-known limitations of the PBE functional in predicting accurate band gaps, we performed additional calculations for the 4-Ab, 4-AB-α-FM–afm, and 4-AB-β-FM–afm nanoribbons using the HSE06 hybrid functional. Figure 10 displays the electronic band structures obtained with both PBE (dashed black lines) and HSE06 (solid red lines) functionals. The results yield qualitatively similar band profiles for both methods. The most significant difference is a shift of the frontier bands away from the Fermi level in the HSE06 calculations, resulting in wider band gaps of 0.43, 1.10, and 1.23 eV for 4-Ab, 4-AB-α-FM–afm, and 4-AB-β-FM–afm systems, respectively. The GGA-PBE functional was employed in this study due to its balance between computational cost and physical accuracy. Although the resulting band gaps should be interpreted as lower limits due to the functional’s inherent underestimation, the fundamental electronic features and trends across the systems are expected to be accurately described.

**Figure 10:**
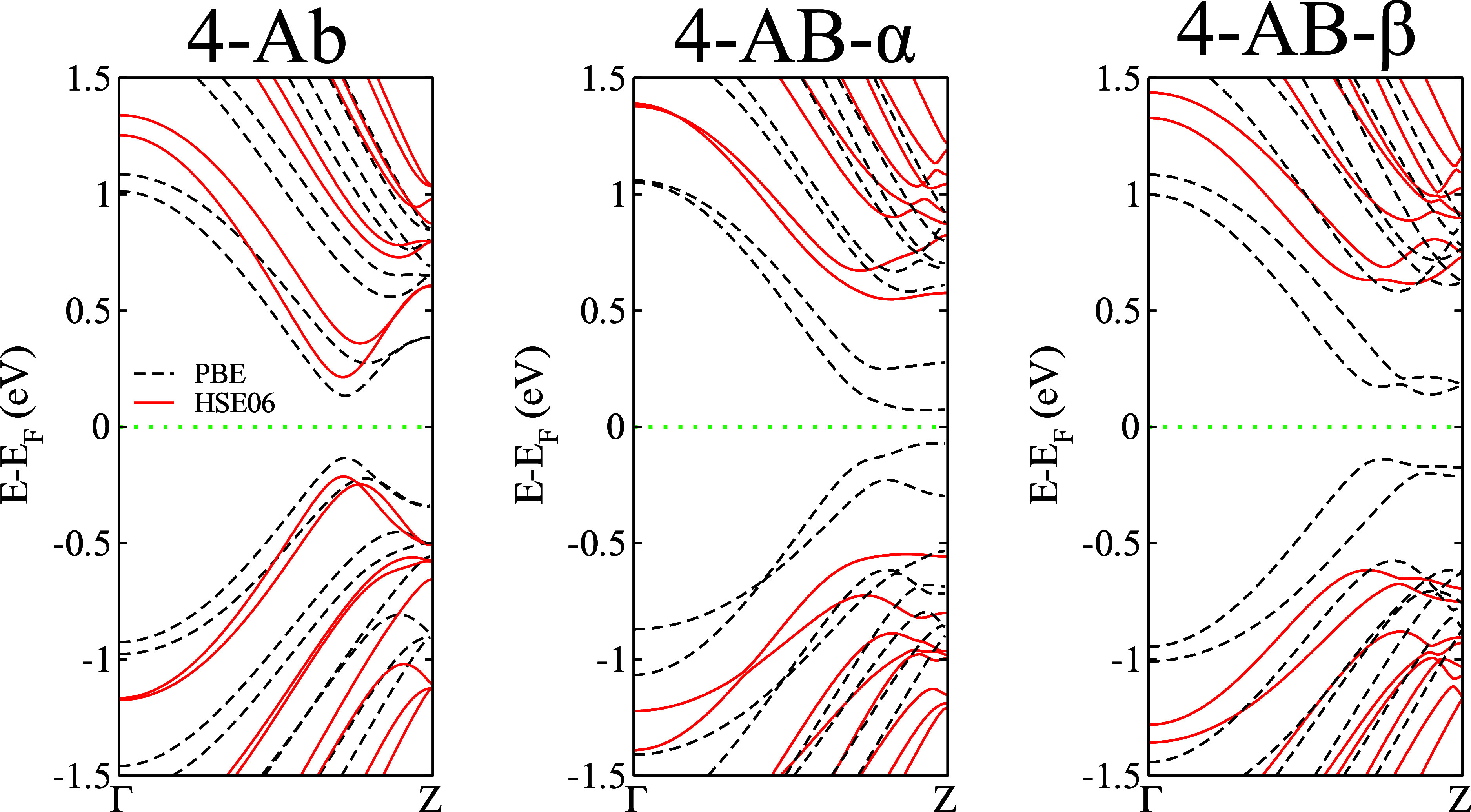
Comparison between the electronic band structures obtained with the PBE (dashed black lines) and HSE06 (solid red lines) simulations for 4-Ab, 4-AB-α-FM–afm, and 4-AB-β-FM–afm systems.

The magnetic moments of the AFM–fm and FM–afm configurations for both α and β alignments are zero, due to the symmetry between spin-up and spin-down charge densities, as evidenced by their band structures and spin density plot (see Figure 9). In contrast, the AFM–afm and FM–fm states exhibit nondegenerate spin bands near the Fermi energy, resulting from noninteger magnetic moments, as shown in Table 1. The FM–fm configurations naturally display higher magnetic moment values as their four edges have the same majority spin, which also explains the greater breaking of band degeneracy near the Fermi level of these states when compared to the AFM–afm cases. This behavior can be confirmed from the band structures of the 4-α-BZGyNR system shown in Figure 9. It is important to note that a zero total magnetic moment in the FM–afm and AFM–afm states does not imply the absence of magnetic ordering. In these configurations, the finite magnetization energy indicates that the spin-polarized states are energetically more stable than the spin-unpolarized states, despite the complete compensation of local magnetic moments and the consequent zero total magnetic moment.

**Table 1:** Total Magnetic Moment in the Unit of μ_B_ for α-BZGyNRs

α-BZGyNRs	AFM–afm-α	AFM–afm-β	FM–fm-α	FM–fm-β
2	0.006	0.054	0.006	0.062
3	0.002	0.011	0.500	0.646
4	0.002	0.029	0.745	0.815
5	0.004	0.061	0.856	0.917
6	0.016	0.091	0.932	0.987
7	0.036	0.120	0.986	1.037
8	0.056	0.139	1.021	1.073

The α-BZGyNRs exhibit stacking-dependent electronic behavior, displaying either a semiconducting or a metallic character, with a high DOS at the Fermi level in some cases. Therefore, controlling the stacking arrangement of α-BZGyNRs is crucial for their potential application in devices. For instance, the AB-α and AB-β stackings are particularly promising for nanoelectronic applications, as they accommodate distinct spin-polarized electronic configurations. All of these spin states occur in nonplanar structures, as discussed in section “Studied Structures”, in contrast to BZGNRs, for which only the planar AB-β configuration exhibits spin polarization.^29^ This is a key advantage of α-BZGyNRs over BZGNRs, as structural distortions do not suppress magnetization in α-BZGyNRs. Although the systems investigated in this study have not yet been synthesized experimentally, previous reports describe the synthesis of various members of the GY family, including multilayer films,^10,12^ multilayer quantum dots,^59,60^ and nanoribbon-like graphdiyne systems.^13^ Therefore, our results are expected to guide future experimental and theoretical efforts aimed at exploring spin-dependent properties in bilayer GY systems.

### Modulation by an External Electric Field

For more flexibility in device design and application, it is highly desirable that the band gap of materials can be tuned as needed. Previous works have reported the modulation of the electronic structure of monolayer zigzag-edge nanoribbons based on graphene and graphyne lattices.^15,19,55,57^ For instance, the AFM state of a monolayer zigzag graphene nanoribbon can undergo a transition from semiconducting to half-metallic under a sufficiently large transverse electric field.^15,55^ A similar effect has been observed in BZGNRs with AB-β stacking^27,61^ under the action of a transverse electric field on the bilayer. Motivated by these findings, we investigated the effect of an externally applied electric field on the electronic properties of the α-BZGyNRs. To this end, we selected the 4-α-BZGyNR-AB-α system in its FM–afm ground state.

Before moving further, let us look at more details on the charge distribution and frontier states of the system in the absence of an external electric field. For a qualitative understanding of the charge distribution, we compute the Δ*q*_i_ = *q*_i_^M‑up^ – *q*_i_^M‑down^, where *q*_i_^M‑up^ and *q*_i_^M‑down^ are the spin-up and spin-down Mulliken charges on the *i*th atom, respectively. Such a quantity gives us an idea of which atoms have the most important contribution to spin polarization. Within the unit cell, we note that the outermost carbon edge atoms from each nanoribbon layer (the hydrogenated ones) carry the largest |Δ*q*_i_| values. From these four atoms, those closer to the center of the system have |Δ*q*_i_| slightly larger than the farther ones: 0.145 electron for the former versus 0.139 for the latter. In the lower panel of Figure 11, we plot the LDOS for the spin-up and spin-down CBM and VBM states in the absence of a field (ϵ = 0). To ease the discussion, we will label these atoms as R1, R2, L1, and L2, with R-L indicating right- or left-hand side edges and 1 and 2 indicating an atom closer to or further from the system’s axis. In this way, atoms L1 and L2 (R1 and R2) are spin-up (spin-down) polarized. When we examine the occupied VBM states, the dominant contribution to the spin-up component comes from the edge of the L1 atom (where the majority spin is spin-up). Likewise, the major contribution from the VBM spin-down component comes from the edge of the R1 atom (for which the majority spin is spin-down). This picture reveals a consistency between the spin polarization of these edges and the spatial distribution of the VBM in the FM–afm state of the 4-α-BZGyNR-AB-α system (illustrated in Figure 9), as the spin-resolved frontier occupied states come from the edge atoms with the largest spin polarization (largest |Δ*q*_i_|) and match their corresponding majority spin orientation. Such a consistency is also observed for the CBM states, as its spin-up component comes mostly from the R2 edge, which has the opposite (spin-down) orientation as the majority spin (it also spreads significantly over R1, which also has majority spin-down). Similarly, the spin-down CBM states come mainly from the L2 edge, which has majority spin-up (it also spreads significantly over L1, which also has majority spin-up). In summary, the distribution of the frontier states in this configuration closely follows aspects of the spin-polarization distribution. Another important aspect to highlight is that spin-up frontier states (both VBM and CBM) spread mostly on the lower layer, while the corresponding spin-down states lie mostly on the upper layer.

**Figure 11:**
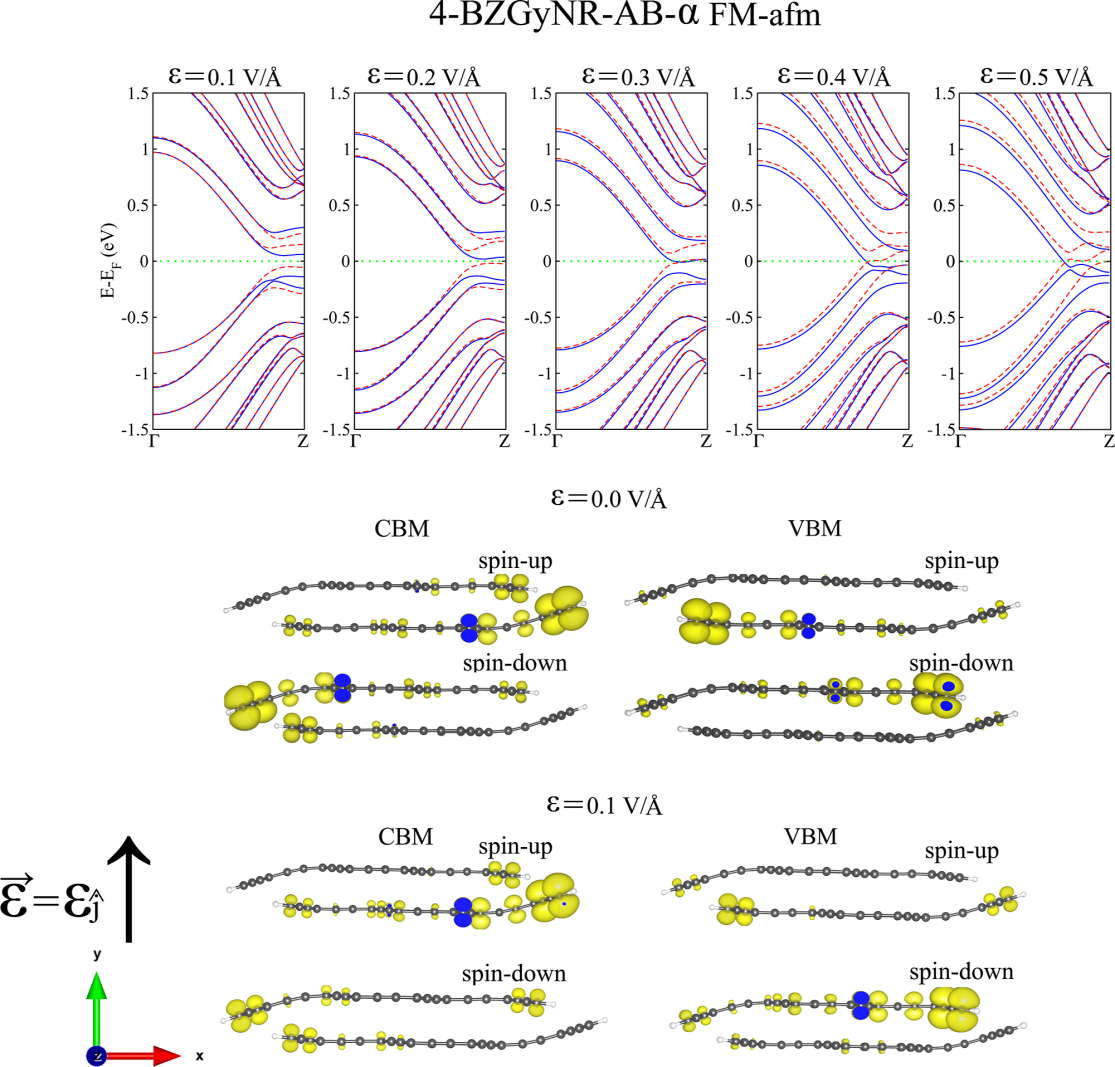
(Upper panel): Electronic band structures of the 4-α-BZGyNR-AB-α in the FM–afm configuration for different external electric fields orthogonal to the bilayer plane. (Lower panel): LDOS for VBM and CBM under electric fields of 0.0 and 0.10 V/Å. In the LDOS plot, the yellow regions represent the electron density distribution, while the blue regions appear due to the cross-section of the surface in the visualization procedure and have no physical meaning.

Let us now consider an external electric field orthogonal to the plane of the bilayer nanoribbon. Without a transverse electric field, the electrostatic potential profiles of both layers are identical. However, upon the application of the field, the electrostatic potential *V* decreases in the direction of the field (E⃗=−∇V) , leading to an increase/decrease in the potential energy *U* (=–*eV*) for the upper/lower layer (Δ*U* = *U*_upper_ – *U*_lower_ = – *e*Δ*V* > 0.0). As we will discuss, this potential difference in the structure of the α-BZGyNR will be directly associated with changes in the band gap, since the energy of the electronic levels localized on the upper layer tends to be shifted upward, while those on the lower layer are shifted downward with respect to the Fermi level.

The electronic band structures under different strengths of the orthogonal electric field are shown in the upper panel of Figure 11. For an electric field of 0.10 V/Å (and for the other nonzero field values considered here), the spin degeneracy around *E*_F_ is split, as expected from symmetry arguments. Note that in the absence of an electric field, a 180° rotation around an axis parallel to the periodic directions and passing by the centroid of the structure swaps the position of the spin-up and spin-down clouds, explaining why this system has spin-degenerate sets of bands. Under a nonzero electric field perpendicular to the ribbons, such a symmetry is broken, as we expect a larger accumulation of electrons in the lower ribbon from the diagrams in the lower panel of Figure 11. To obtain an additional qualitative illustration of this, we computed the total Mulliken charge of the lower layer, which increases by 0.047 electron relative to the configuration with no field, consistent with the previous discussion on the potential profile.

In the ϵ = 0.10 V/Å case, the spin-down VBM is shifted upward along the energy axis. This occurs because the corresponding zero-field states lie mostly on the R1 edge of the upper layer, and the field orientation is such that it tends to remove charges from the upper ribbon, consequently shifting the spin-down VBM to higher energies (toward *E*_F_). Looking at the LDOS for this ϵ = 0.10 V/Å state (lower panel of Figure 11), it has a profile similar to that in the absence of a field but slightly more delocalized over the upper ribbon width. The same trend is observed for the spin-down CBM, which is also on the upper layer in the zero field, shifts toward higher energies, and spreads more homogeneously over the entire upper layer. On the other hand, both the VBM and CBM spin-up states shift to lower energy values, as they lie mostly on the bottom layer (which becomes the acceptor sector of the system as we apply the electric field). As a result of these energy shifts of the frontier levels, the gap of the system is reduced from 176 meV (no field) to 100 meV (ϵ = 0.10 V/Å), and the gap is now between a spin-down (VBM) and a spin-up (CBM) state. For higher field values, these effects are reinforced, the gap further narrows to 37 meV for ϵ = 0.20 V/Å, and the system experiences a semiconductor-to-metal transition at ϵ = 0.30 V/Å. Further higher fields promote the crossing of an additional band at the *E*_F_, as observed, for instance, for ϵ = 0.50 V/Å.

We now move to the case in which the field is still orthogonal to the system axis but parallel to the average molecular planes. Figure 12 presents the electronic band structures for different values of the applied electric field in the 4-α-BZGyNR-AB-α. In this figure, we also reproduce the LDOS for the spin-up and spin-down states for the zero-field case and for ϵ = 0.10 V/Å. One feature that is similar to the previous orthogonal field case is that spin degeneracy for the bands around *E*_F_ is split for nonzero field values. Here, the field points from the spin-up to the spin-down polarized edges, so that now charges tend to accumulate on the edges of the L1 and L2 atoms, making the spin-up/spin-down asymmetry even more explicit than in the orthogonal field configuration. In addition, this different field geometry induces different modifications in the system’s band structure, as we will now discuss.

**Figure 12:**
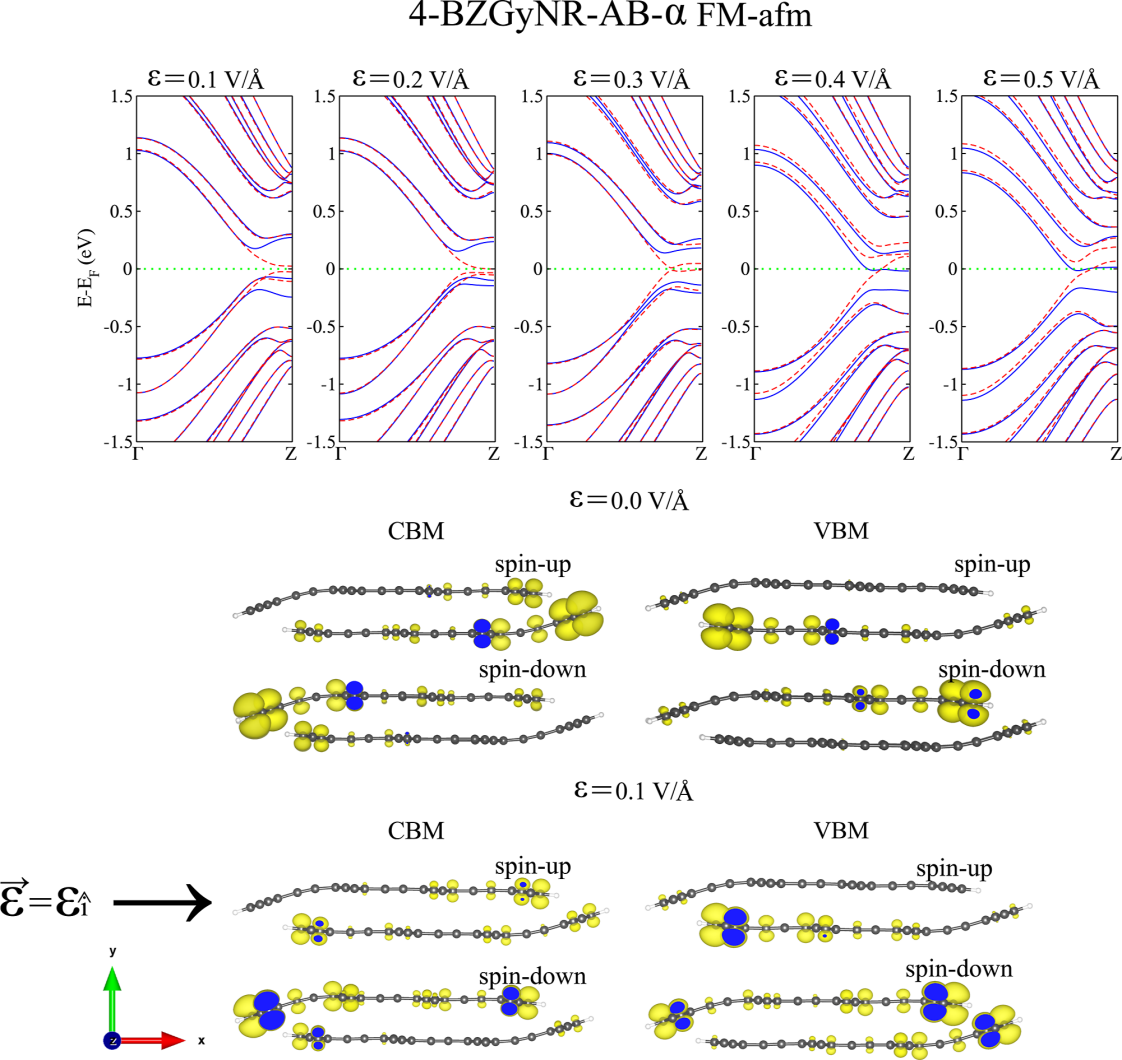
(Upper panel): Electronic band structures of the 4-α-BZGyNR-AB-α in the FM–afm configuration for different external electric fields parallel to the molecular plane of the bilayer. (Lower panel): LDOS for VBM and CBM under electric fields of 0.0 and 0.20 V/Å. In the LDOS plot, the yellow regions represent the electron density distribution, while the blue regions appear due to the cross-section of the surface in the visualization procedure and have no physical meaning.

We recall that the spin-up/spin-down VBM state is mainly distributed over the L1/R1 edge. So, according to their position along the width and relative to the field direction, we expect that the spin-up VBM tends to shift toward lower energies (as its occupation is expected to increase), whereas the opposite trend is expected for the spin-down VBM. This is exactly what we observe for the spin-down VBM for the system under an electric field of 0.10 V/Å, as shown in the upper panel of Figure 12, while the spin-up somehow shifts to a much smaller extent. This is reflected in the LDOS profile of the spin-up and spin-down VBM (see the lower panel from Figure 12), as the spin-down state now shows significant amplitude over the leftmost L2 edge, at the same time that only mild modifications are observed in the spatial profile of the spin-up state. The spin-down VBM further starts to show amplitude over the lower nanoribbon, especially over the R2 edge, as it approaches the Fermi level.

When it comes to the bottom conduction states, the spin-up CBM is mostly over the R2 edge for zero field, tending to shift to higher energy values in nonzero fields, as in the ϵ = 0.10 V/Å field case. In fact, the gap for spin-up states under this field value is enlarged to 247 meV, compared to the 176 meV value for the field-free system. The LDOS for this state changes from a dominance of the R1 and R2 edges to a profile with a somewhat more equilibrated contribution for the L1 (compared to the R1) edge. For the spin-down CBM, mostly located at the L2 edge, the expected trend in nonzero fields is that these levels accept electrons and shift down in energy, as observed for the ϵ = 0.10 V/Å field. As a result, the spin-down gap narrows (from 176 meV at no field to 51 meV for ϵ = 0.10 V/Å). In this system, the overall VBM and CBM states have the same spin component, similar to what occurs for the monolayer counterpart.^15^ Due to this narrow gap, the spin-down VBM/CBM states start to hybridize so that the CBM starts to show amplitude over the R1 edge (a feature from the VBM). These effects are reinforced for ϵ = 0.20 V/Å, where the system closes the spin-down gap and becomes half-metallic (228 meV gap for spin-up states). For ϵ = 0.40 V/Å, the spin-up CBM exhibits a strongly different profile compared to the zero-field case, so that this level for even higher field values acquires a different trend and shifts down, turning the spin-up conduction band into a semifilled branch.

These findings demonstrate that the electronic properties of the system can be effectively tuned by applying an external electric field. We notice two distinct behaviors: (1) when the external electric field is orthogonal to the bilayer plane, a semiconductor-to-metallic transition occurs for certain field values; (2) when the field is parallel to the molecular plane, first a semiconductor-to-half-metallic transition occurs (for the ϵ of 0.20 V/Å), and then a transition to metallic occurs for the 0.40 V/Å field. These results may be of particular interest for the development of nanoscale electronic devices.

### Summary and Concluding Remarks

In summary, we presented a theoretical investigation of the electronic properties of bilayer nanoribbons based on the α-graphyne lattice. Four stacking configurations were considered: AA, Ab, AB-α, and AB-β, which result in nonplanar atomic structures, with the AB-α stacking mode exhibiting significant edge curvature. We found that the electronic behavior is strongly dependent on the stacking mode, displaying either metallic or semiconducting character, with the band gap modulated by ribbon width. Unlike bilayer graphene-based ribbons, the nonplanar geometry of the α-BZGyNRs does not suppress magnetism. In particular, the AB-α and AB-β stackings support distinct spin-polarized electronic distributions. The electronic properties of the SP states are shown to depend on both interlayer and intralayer magnetic alignments, as well as on ribbon width. We identified SP configurations with metallic, semiconducting, and half-metallic character. Furthermore, we examined the influence of an external electric field on the electronic properties of a representative system in its ground-state magnetic configuration. The results revealed that applying an electric field allows for the adjustment of the band structure, including a semiconductor-metal or semiconductor-half-metal transition depending on the direction of application and intensity of the electric field. Overall, our findings highlight the rich spin-dependent electronic behavior of α-BZGyNRs and demonstrate their potential for modulation via structural and external parameters. These insights may stimulate future experimental and theoretical efforts aimed at exploring and controlling spintronic functionalities in graphyne-based bilayer systems.

## Supplementary Material



## Notes

Energy difference between each spin-polarized (SP) and the corresponding nonpolarized (NP) state for 2-AA, AB-α, and AB-β systems as a function of the α-BZGyNRs width (PDF)
