# Supplementary material for: Spin-Dependent Electronic Properties of Bilayer α‑Graphyne Zigzag Nanoribbons: A Density Functional Theory Study
Source: ACS Omega. 2026 Jul 1;11(27):40266–82. doi: 10.1021/acsomega.6c02506 (PMC13382674; doi:10.1021/acsomega.6c02506)
Supplement: Supplementary file 1 [file ao6c02506_si_001.pdf]

Supporting Information for:

Spin-dependent electronic properties of bilayer α-graphyne zigzag nanoribbons: a density functional theory study

Maycon Ericles Macedo Barros [1],Eduardo Costa Girão [2], Vincent Meunier [3], and Paloma Vieira Silva*[2],

[1]Programa de Pós-graduação em Física, Universidade Federal do Piauí, Teresina, Piauí, 64049-550, Brazil

[2]Departamento de Física, Universidade Federal do Piauí, Teresina, Piauí, CEP 64049-550, Brazil

[3]Department of Engineering Science and Mechanics, , State College, Pennsylvania State University, USA

**2-AA**	**$\Delta E = E_{NP} - E_{AFM\text{-}afm}$ (meV)**
AFM-afm	3,737

Table S1: Energy difference between the AFM-afm and non-polarized state for the 2-α-BZGyNR-AA system.

**States for AB-ɑ stacking**	**$\Delta E = E_{NP} - E_{SP}$ (meV)**						
	**2**	**3**	**4**	**5**	**6**	**7**	**8**
AFM-afm	16.423	32.556	41.403	45.571	46.758	48.610	48.935
AFM-fm	…...	…….	28.159	37.633	44.331	47.697	49.845
FM-afm	22.982	38.052	48.898	53.641	56.915	59.396	60.896
FM-fm	…...	13.264	28.468	38.019	43.582	49.391	53.672

Table S2: Energy difference between each spin-polarized (SP) and the corresponding non-polarized (NP) state for all the α-BZGyNRs systems with AB-ɑ stacking.

States for AB-β stacking	$\Delta E = E_{NP} - E_{SP}$ (meV)						
	**2**	**3**	**4**	**5**	**6**	**7**	**8**
AFM-afm	5.727	26.013	35.537	41.307	44.783	48.430	51.112
AFM-fm	…...	8.990	28.002	38.664	44.861	50.091	53.289
FM-afm	32.049	51.885	63.564	69.730	73.255	76.028	77.649
FM-fm	…...	25.750	42.755	53.226	60.184	65.524	69.198

Table S3: Energy difference between each spin-polarized (SP) and the corresponding non-polarized (NP) state for all the α-BZGyNRs systems with AB-β stacking.
